# Supplementary material for: Cigarette smoke increases susceptibility to infection in lung epithelial cells by upregulating caveolin-dependent endocytosis
Source: PLoS One. 2020 May 21;15(5):e0232102. doi: 10.1371/journal.pone.0232102 (PMC7241776; doi:10.1371/journal.pone.0232102)
Supplement: S1 Raw images — (PDF) [file pone.0232102.s001.pdf]

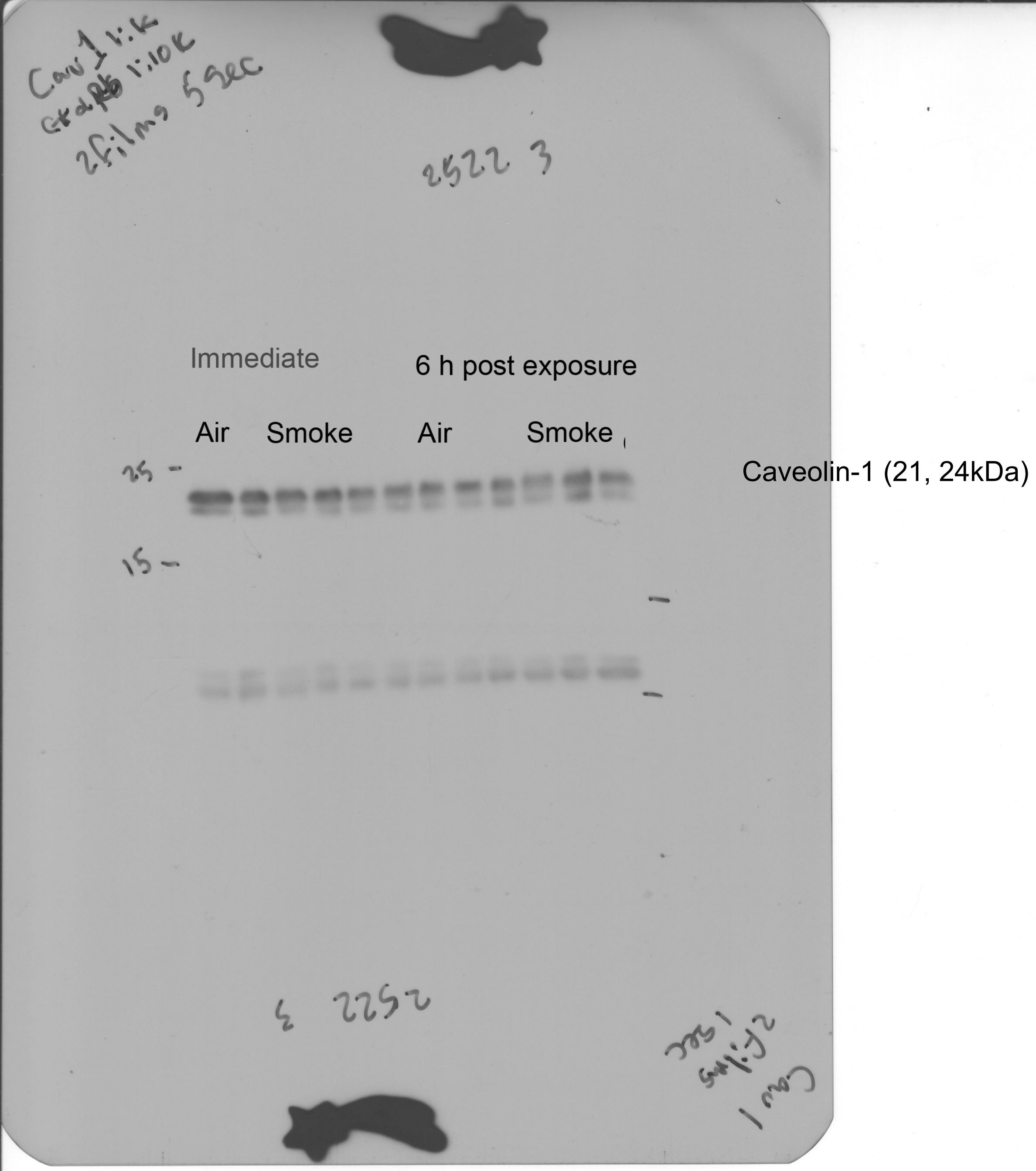
Immediate
6 h post exposure
Air
Smoke
Air
Smoke
25 -
15 -
Caveolin-1 (21, 24kDa)
2522 3

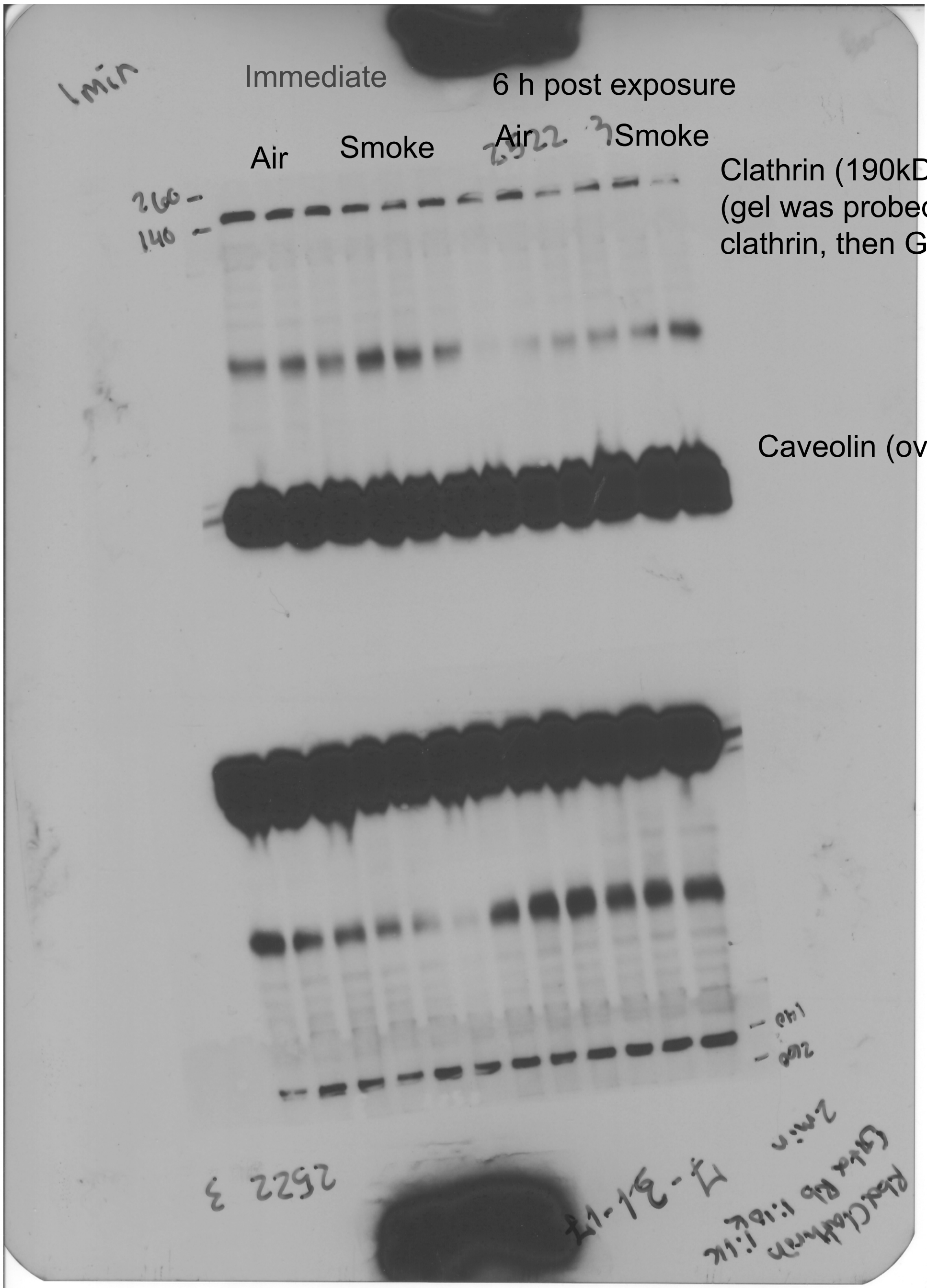

Clathrin (190kDa)
(gel was probed for caveolin first, then clathrin, then GAPDH)

Caveolin (overexposed)

Same blot longer exposure

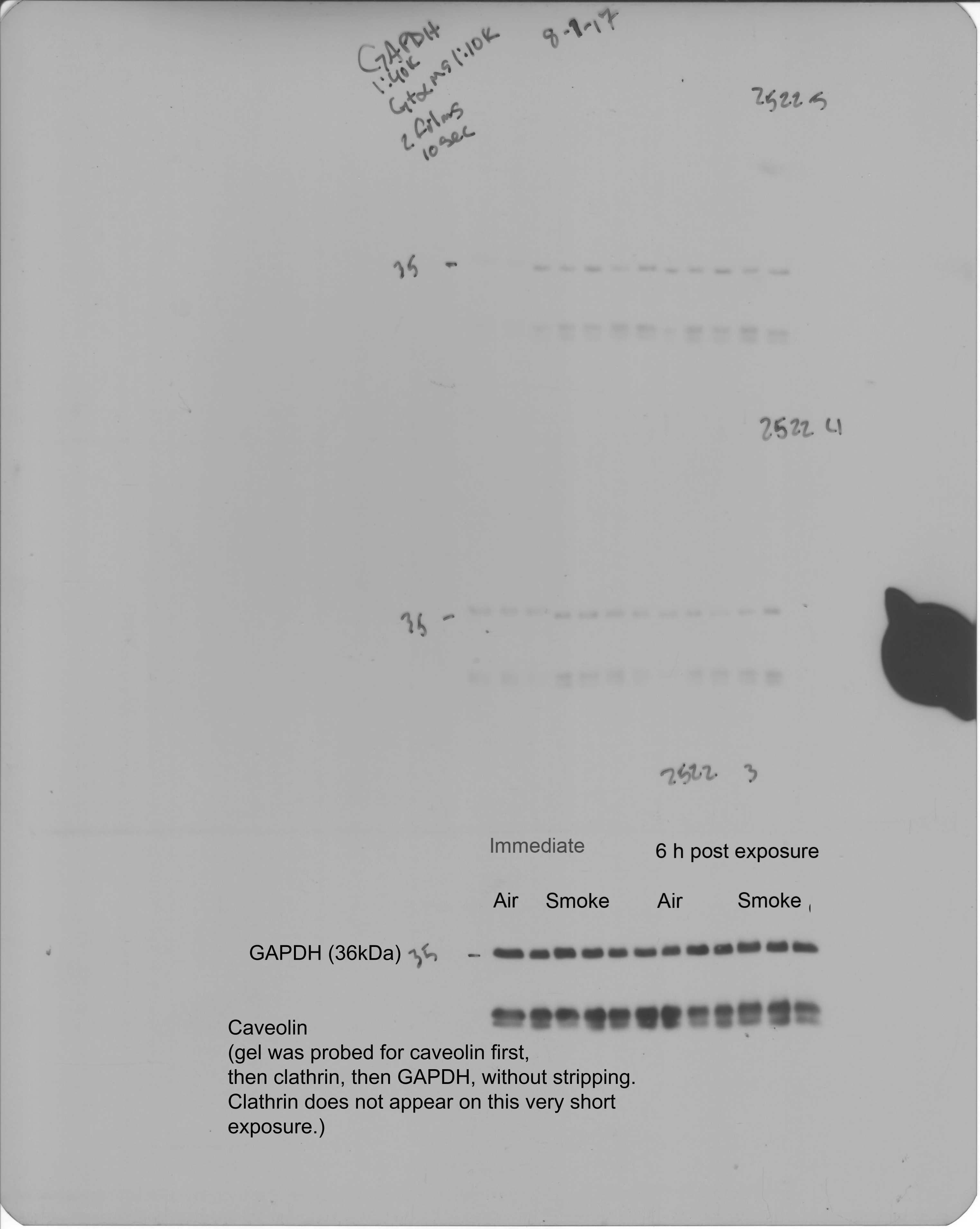
GAPDH
1:40K
8-9-17
2522 5
35 -
2522 4
35 -
2522 3
Immediate
6 h post exposure
Air
Smoke
Air
Smoke
GAPDH (36kDa) 35 -
Caveolin
(gel was probed for caveolin first,
then clathrin, then GAPDH, without stripping.
Clathrin does not appear on this very short
exposure.)
