# Supplementary material for: Cigarette smoke increases susceptibility to infection in lung epithelial cells by upregulating caveolin-dependent endocytosis
Source: PLoS One. 2020 May 21;15(5):e0232102. doi: 10.1371/journal.pone.0232102 (PMC7241776; doi:10.1371/journal.pone.0232102)
Supplement: Attachment — Submitted filename: Response to reviewers PONE-D-19-26455.docx [file pone.0232102.s002.docx]

**Original reviewers’ comments and responses**

Reviewer #2: The study by Duffney et al., investigates the effect of cigarette smoke on endocytosis in primary human small airway epithelial cells (SAEC) The work is interesting as it may shed light into the mechanisms responsible for enhanced susceptibility to viral infections in smokers. Several technical points need to be addressed, specifically:
Major points:
- Fig. 2A, it appears that very little I:C internalization occurs in air-exposed SAEC cells, unlike in panel B; please clarify the discrepancy. Fluorescence appears to increase over the time course in smoke-treated cells, despite any unbound ligand being washed away from the cells, thus preventing further internalization; what is the explanation for this behaviour?

*The pictures in 2A were taken with a simpler monochrome camera, the images in 2B were taken with a confocal microscope, and the magnification is different, we think the difference is simply due to sensitivity. Also, the labeled poly(I:C) was left on the cells for the indicated time (2, 6 or 19 hours), it was not washed away until just before imaging, so there is no increased uptake in the absence of the label. We apologize for any confusion.*

- Fig. 2B, it is not clear at what interval during the time course experiment “the representative images” were taken (see also the comment above).

*The photos in 2B were taken 6 hours after treatment with poly I:C which represents the first time point where there was noticeable difference between air and smoke exposed cells in the uptake of poly I:C. We have clarified this in the figure legend.*

- Fig. 2C, what do the authors mean by “three strains of SAEC” tested?

*We obtained SAEC from three different donors, we refer to these as “strains” (since they are not clonal, we prefer to call them cell strains rather than cell lines). We have clarified in the legend that each pair of points represents cells from a different donor.*

- Fig. 3A, from the black and white pictures it can be concluded that only the bright fluorescent cells have taken up the ligand, while the remaining cells, showing just background fluorescence have not. Thus it appears that more BSA is found in smoke-treated SAECs, which is in disagreement with Fig. 3B and the overall interpretation of this experiment. Please show representative fluorescence microscopy images for transferrin uptake as well.

*We appreciate the reviewer comment. We have included new confocal images and computer-assisted cell counting to show the uptake of BSA into the cells (new figure 3A and B). The highly fluorescent cells may represent a subset of SAEC that take up higher levels of BSA.*

- Fig. 4A, only a few cells displaying bright fluorescence are shown, while the surrounding cells don’t appear to have taken up CtxB. Please include 20x magnification images to demonstrate that the ligand uptake is not restricted to a limited number of cells.

*We apologize for the confusion. We repeated the experiment and took new images with the confocal scope, and have included computer-assisted cell counts as well (New figures 4A and 4B.)*

- Fig. 5A is not convincing at all. The image shows very crippled cells, or cell debris. What is the purpose of alternating black and white/colour images?

*We appreciate the reviewer’s comment. We found no difference in the cell morphology with the addition of fluorescent dextran in either group and have seen previously that exposure to this level of cigarette smoke does not alter cell viability (Duffney et al. 2018 AJP- lung). Since it was difficult to quantify fluorescence in images of the dextran treated cells, we performed flow cytometry to quantitate fluorescence in the cells (Figure 5B). Serial images of live cells were acquired using a monochromatic camera and we did not apply a false color rendering; fixed cells were imaged with a color system.*

- Fig.6 assessing the overall intracellular levels of caveolin and clathrin is not very meaningful to draw conclusions on endocytosis; rather, the analysis should focus on the plasma membrane pool of these proteins, by subcellular fractionation or fluorescence microscopy.

*We appreciate the comment from the reviewer. Caveolin is an integral membrane protein thus fractionation would not be a relevant endpoint for caveolin activation. Previous work has shown that disease states like IPF and lung cancer, which have altered caveolin-mediated endocytosis occur with changes in the overall caveolin protein levels (Lu, Z. et al. Cancer Cell. 2003, and Wang, X. et al. Journal of Experimental Medicine. 2006). Thus, we believe monitoring total caveolin protein levels is a meaningful endpoint for measuring alteration of caveolin-mediated endocytosis.*

- Fig. 7 is very problematic as there is hardly any IAV infection of SAECs in controls. IAV infection occurs via clathrin-mediated endocytosis in most susceptible cell lines, although caveolin may also be involved in other cell types. Clarification on the mechanism of IAV entry in SAECs under normal conditions is necessary. The conclusion that enhanced IAV infection in smoke-exposed SAECs is due to upregulated entry is not sustained by the data shown in this figure, as clearly, other steps of the IAV life cycle could be affected in this experimental setting.

*The reviewer’s concerns are important. Figure 7A was performed at a very low MOI in order to see differences that obscured at higher MOI where the amount of virus can saturate the cells. However, we agree we are measuring the total life cycle, including initial uptake, replication, and uptake of newly propagated virus. To add confirmation that the increased caveolin uptake was responsible for increased viral replication, we infected smoke-exposed SAECs with a higher titer of virus particles and treated them with filipin III, a caveolin inhibitor. Since filipin III significantly reduced the number of virus-infected cells at 24 h, we conclude that at least part of the reason for increased replication is enhanced caveolin-mediated uptake.*

Minor:
Explain abbreviations when first used (see SAEC, page 4, line 88). *We have gone through and defined all abbreviated terms after their first use.*
Explain PIC and FIC abbreviations in Fig. 1 caption. *We have defined the abbreviations of FIC and PIC in the figure 1 legend.*
Discussion could be shortened by avoiding unnecessary repetition of results interpretation*. We reworded parts of the discussion to improve flow and comprehension.*
