# Supplementary material for: Cigarette smoke increases susceptibility to infection in lung epithelial cells by upregulating caveolin-dependent endocytosis
Source: PLoS One. 2020 May 21;15(5):e0232102. doi: 10.1371/journal.pone.0232102 (PMC7241776; doi:10.1371/journal.pone.0232102)
Supplement: Attachment — Submitted filename: Review_AJ_Cigarette smoke increases susceptibility to infection in lung epithelial cells by upregulating caveolin.docx [file pone.0232102.s003.docx]

**„Cigarette smoke increases susceptibility to infection in lung epithelial cells by upregulating caveolin-dependent endocytosis”**

Parker F Duffney and colleagues present a manuscript that investigates the mechanism by which lung epithelial cells exposed to CS get more susceptible to virus infections. They identified the caveolin-dependent endocytosis as the main path for viruses to enter epithelial cells of smokers.

Results cover following aspects:

- Evaluation of the functionality of the FITC-labeled poly I:C compared to the unlabeled poly I:C in SAEC – Elisa of involved cytokines
- Effect of CS on the uptake of poly I:C
- Involvement of different endocytosis pathways to better understand the uptake of virus particle by epithelial cells.

Figure 3:

A. Include bright field to have a better clue where the labeled-BSA (BSA-AF594) is located in the cell after uptake.

Authors do not mention how long cells were incubated with the dye and when the readout was (in the legend they refer to the methods part and in the methods part they say “cells were then treated ….for the indicated times”. Important to know whether authors, similar to the already published data on uptake of FITC-labeled BSA look at the cells after 60min or whether they wait longer. Fluorescent signal in reference nr. 20 shows a punctated localization which differs from the presented pictures here in Figure 3 (due to timing?)

B. Specify the y-axis more – (%) BSA-uptake

Figure 4:

A. CtxB is also here a labeled one, please keep the same name in case of similar quantifications (CtxB-AF594 like in 4B), same for Figure C

D: the 63 magnification of the CS/LacCer picture shows a stronger

background signal than the one of the control. Users should take a

magnicication of a cell that is rather far away from the other positive cells

to avoid misunderstanding of exposure differences.

Figure 5:

Line 281/282: cellular fluorescence was difficult to quantify using automated image analysis… (First: there is no need to rely on only automated image analyzer, authors could optionally quantify single images, second: no need to mention this in the text since the picture is convincing).

Figure 6:

Authors should try to show the blots such that the bands are under each other. Labeling of the samples is not straight forward (immediate?), it would be rather better if authors used “short exposure” and “6h exposure”. In order to prove that there are no changes in clathrin and caveolin upon CS exposure, authors should add the quantification of band intensities. In addition, the second strain shows a slight reduction in both samples, the one immediately and the one 6h post CS exposure, how do authors deal with this.

Figure 7: very nice experiment, that shows the specificity of the virus uptake. It is necessary to exclude any effects of filipin III on the previously described pathways. Meaning, authors should include the experiments of labeled BSA and labeled CtxB with fillipin III and show that this does not affect the uptake of these compounds to finally confirm the uptake of the WSN virus exclusively by caveolin-dependent endocytosis.

Notes:

Line 279: a common marker OF macropinocytosis…
