# Supplementary material for: Cigarette smoke increases susceptibility to infection in lung epithelial cells by upregulating caveolin-dependent endocytosis
Source: PLoS One. 2020 May 21;15(5):e0232102. doi: 10.1371/journal.pone.0232102 (PMC7241776; doi:10.1371/journal.pone.0232102)
Supplement: Attachment — Submitted filename: Response_Duffney et al 2-21-20a.docx [file pone.0232102.s004.docx]

21 February 2020

Dr. Jorg Heber

Editor, PLOS ONE

Dear Dr. Heber,

Thank you for your continued consideration of our manuscript PONE-D-19-26455, “Cigarette Smoke Increases Susceptibility to Infection in Lung Epithelial Cells by Upregulating Caveolin-Dependent Endocytosis“. We have responded to the comments of the reviewers and editor as shown in the attached response and the annotated manuscript. We believe the changes have clarified and improved the manuscript and look forward to its publication in PLOS ONE.

I confirm that all authors have reviewed the work, and this work is not under consideration elsewhere.

Sincerely on behalf of the authors,

Patricia J. Sime

Professor and Chair, Department of Internal Medicine

Virginia Commonwealth University

1101 East Marshall Street

1^st^ Floor Suite 1-030

Richmond VA 23298

**Response to reviewer comments**

Figure 3:

A. Include bright field to have a better clue where the labeled-BSA (BSA-AF594) is located in the cell after uptake.

Authors do not mention how long cells were incubated with the dye and when the readout was (in the legend they refer to the methods part and in the methods part they say “cells were then treated ….for the indicated times”. Important to know whether authors, similar to the already published data on uptake of FITC-labeled BSA look at the cells after 60min or whether they wait longer. Fluorescent signal in reference nr. 20 shows a punctated localization which differs from the presented pictures here in Figure 3 (due to timing?)

We appreciate the reviewer comment. Unfortunately, we did not take brightfield images for of the cells in figure 3A. Despite this, we think the image sufficiently shows labeling consistent with both surface and intracellular staining. We updated the methods (lines 146-149) to clarify that for the confocal images, cells were loaded with ligand for 30 min at 4°C and then incubated at 37°C for 30 min before fixing and visualizing. While other studies have seen distinct puncta staining at 60 minutes, this shortened incubation time could explain why we see both surface and internal labeling.

B. Specify the y-axis more – (%) BSA-uptake

We have changed the axis to specify the uptake in Figure 3B is BSA uptake (New graph for figure 3B).

Figure 4:

A. CtxB is also here a labeled one, please keep the same name in case of similar quantifications (CtxB-AF594 like in 4B), same for Figure C

We thank the reviewer for the comment and have adjusted the labeling for consistency throughout the figure (Line 237-242). The AF594-CtxB was used for the confocal imaging and subsequent image quantification. AF488-CtxB was used for flow cytometry labeling. We adjusted the labeling of Figures 4B and 4C to clarify this.

D: the 63 magnification of the CS/LacCer picture shows a stronger

background signal than the one of the control. Users should take a

magnicication of a cell that is rather far away from the other positive cells

to avoid misunderstanding of exposure differences.

Both images were acquired at the same exposure settings, and we adjusted the contrast to see clearly the label uptake in untreated cells. Because the smoke-exposed cells took up so much more label, this resulted in overexposed images of smoke-exposed cells. Unfortunately, these were semi-permanent mounts only and are no longer available to re-image. We have adjusted the contract on the images presented (using the same settings for both) to reduce the background, this unfortunately reduces the ability to see the label uptake in the control cells but we think the images will still be suitable for publication.

Figure 5:

Line 281/282: cellular fluorescence was difficult to quantify using automated image analysis… (First: there is no need to rely on only automated image analyzer, authors could optionally quantify single images, second: no need to mention this in the text since the picture is convincing).

We appreciate the feedback from the reviewer. We removed the sentence mentioning the difficulties of image analysis with dextran and highlight that similar suppression of dextran uptake was seen when quantitating smoke exposed cells by flow cytometry (Line 525-524).

Figure 6:

Authors should try to show the blots such that the bands are under each other. Labeling of the samples is not straight forward (immediate?), it would be rather better if authors used “short exposure” and “6h exposure”. In order to prove that there are no changes in clathrin and caveolin upon CS exposure, authors should add the quantification of band intensities. In addition, the second strain shows a slight reduction in both samples, the one immediately and the one 6h post CS exposure, how do authors deal with this.

We have gone back and adjusted the western blot figure to line up the bands and make the labeling more clear for the reader. We have also performed densitometry on all the blots from all the strains and found no consistent or significant difference in the expression of clathrin or caveolin in our three strains as a whole. This data is included in new figure 6. While one strain did appear to show significantly decreased protein levels of caveolin (but not clathrin) in the smoke exposed cells, this would not explain why caveolin dependent uptake is enhanced in smoke exposed cells and this finding is not consistent in the other strains tested. Thus we feel that overall, the data does not support the idea that endocytosis changes due to smoke exposure are related to changes in the level of endocytosis proteins (either clathrin or caveolin).

Figure 7: very nice experiment, that shows the specificity of the virus uptake. It is necessary to exclude any effects of filipin III on the previously described pathways. Meaning, authors should include the experiments of labeled BSA and labeled CtxB with fillipin III and show that this does not affect the uptake of these compounds to finally confirm the uptake of the WSN virus exclusively by caveolin-dependent endocytosis.

We agree with the reviewer that showing that showing the effect of filipin III on the uptake of BSA and CtxB would help to strengthen the findings of the figure. However, due to a recent relocation of the lab, the smoke exposure system is temporarily unavailable to perform additional experiments. However, there has been much research on the uptake of BSA and CtxB and it has been shown in numerous studies that BSA uptake utilizes a clathrin dependent mechanism (1-5). The inhibitor, filipin III, has been shown to disrupt caveole formation (6, 7) and CtxB is a common marker of lipid rafts (8-10) and has been shown to be inhibited by filipin III (11, 12). We have added additional information on this topic at lines 361 and 402-404. Despite the inability to perform additional experiments we believe this paper still holds value to the scientific community in its current form.

Notes:

Line 279: a common marker OF macropinocytosis…

This has been fixed.

1.	Gekle M, Mildenberger S, Freudinger R, Schwerdt G, Silbernagl S. Albumin endocytosis in OK cells: dependence on actin and microtubules and regulation by protein kinases. Am J Physiol. 1997;272(5 Pt 2):F668-77.

2.	Lambot N, Lybaert P, Boom A, Delogne-Desnoeck J, Vanbellinghen AM, Graff G, et al. Evidence for a clathrin-mediated recycling of albumin in human term placenta. Biol Reprod. 2006;75(1):90-7.

3.	Margarucci L, Monti MC, Fontanella B, Riccio R, Casapullo A. Chemical proteomics reveals bolinaquinone as a clathrin-mediated endocytosis inhibitor. Mol Biosyst. 2011;7(2):480-5.

4.	Uriarte SM, Jog NR, Luerman GC, Bhimani S, Ward RA, McLeish KR. Counterregulation of clathrin-mediated endocytosis by the actin and microtubular cytoskeleton in human neutrophils. Am J Physiol Cell Physiol. 2009;296(4):C857-67.

5.	Yumoto R, Nishikawa H, Okamoto M, Katayama H, Nagai J, Takano M. Clathrin-mediated endocytosis of FITC-albumin in alveolar type II epithelial cell line RLE-6TN. Am J Physiol Lung Cell Mol Physiol. 2006;290(5):L946-55.

6.	Rothberg KG, Heuser JE, Donzell WC, Ying YS, Glenney JR, Anderson RG. Caveolin, a protein component of caveolae membrane coats. Cell. 1992;68(4):673-82.

7.	Rothberg KG, Ying YS, Kamen BA, Anderson RG. Cholesterol controls the clustering of the glycophospholipid-anchored membrane receptor for 5-methyltetrahydrofolate. The Journal of cell biology. 1990;111(6 Pt 2):2931-8.

8.	Fujinaga Y, Wolf AA, Rodighiero C, Wheeler H, Tsai B, Allen L, et al. Gangliosides that associate with lipid rafts mediate transport of cholera and related toxins from the plasma membrane to endoplasmic reticulm. Mol Biol Cell. 2003;14(12):4783-93.

9.	Chinnapen DJ, Chinnapen H, Saslowsky D, Lencer WI. Rafting with cholera toxin: endocytosis and trafficking from plasma membrane to ER. FEMS Microbiol Lett. 2007;266(2):129-37.

10.	Le PU, Nabi IR. Distinct caveolae-mediated endocytic pathways target the Golgi apparatus and the endoplasmic reticulum. J Cell Sci. 2003;116(Pt 6):1059-71.

11.	Orlandi PA, Fishman PH. Filipin-dependent inhibition of cholera toxin: evidence for toxin internalization and activation through caveolae-like domains. The Journal of cell biology. 1998;141(4):905-15.

12.	Nichols BJ, Kenworthy AK, Polishchuk RS, Lodge R, Roberts TH, Hirschberg K, et al. Rapid cycling of lipid raft markers between the cell surface and Golgi complex. The Journal of cell biology. 2001;153(3):529-41.
